# A Prospective Quality Improvement Initiative to Address the Barriers to Postpartum Tubal Ligation Among Multiparous Women

**DOI:** 10.7759/cureus.29641

**Published:** 2022-09-27

**Authors:** Avir Sarkar, Sivaranjani Panneer, Vidhi Vanya, Isha Wadhawan

**Affiliations:** 1 Obstetrics and Gynecology, Employees' State Insurance Corporation (ESIC) Medical College and Hospital, Faridabad, Faridabad, IND; 2 Obstetrics and Gynecology, All India Institute of Medical Sciences, Kalyani, Kalyani, IND

**Keywords:** developing countries, family planning, low resource countries, contraception, quality improvement, postpartum tubal ligation

## Abstract

Background: Unmet need for postpartum tubal ligation (PPTL) is still high in low-middle income countries. The commonly observed barriers are issues with the consent forms, non-availability of operation theater, lack of knowledge, patient desire for more children, partner opposition and social non-acceptance. Considering the unmet need and the barriers, this quality improvement (QI) initiative was conducted to increase the frequency of PPTL among multiparous women by 50% from baseline.

Materials and methods: This QI was conducted at a tertiary care teaching hospital over a period of 18 months. The study consisted of three phases. Baseline observations during the pre-intervention phase over six months, intervention phase consisting of three Plan-Do-Study-Act (PDSA) cycles over 12 months and post-intervention surveillance phase over a period of three months. In the first PDSA cycle, hospital and provider barriers were focused on. The patient barrier was addressed in the second cycle. The barrier at the level of partner and family members was addressed in the third cycle.

Results: The baseline prevalence of PPTL in the studied population over a period of six months was 30.2%. After the first PDSA cycle, the prevalence of PPTL performed increased from 30.2% to 49.5%. The prevalence increased to 68.8% after the second cycle. A further increase to 74.4% was observed after the third intervention. There was a satisfactory continuation rate of 72% at the end of the post-intervention follow-up phase conducted over four months.

Conclusion: This QI initiative proved effective and sustainable over time. There was continuous motivation among the service providers to educate and counsel women for PPTL. Ultimately, we were able to address the low prevalence of PPTL through minor modifications in our hospital strategies.

## Introduction

Contraception among multiparous women is the need of the hour [1,2]. Tubal ligation is a safe and effective method of contraception for those who have completed childbearing. Tubal ligation is performed following 10% of all deliveries and its frequency is much higher after Cesarean section [3,4]. Unmet needs for postpartum tubal ligation (PPTL) are still high in low-middle income countries (LMIC). Studies have reported that 31% to 48% of requests for PPTL remained unmet [5]. In developing countries with low resource settings, there are much more barriers for women who desire tubal ligation. A study conducted to address the barriers to PPTL in multiparous women stated that the barriers exist at all levels like the hospital system, provider, patient, family, and peer group [6]. The barriers noticed in the study were issues with the process of consent, lack of knowledge, patient desire for more children, sub-optimal counseling, partner opposition, and social non-acceptance [6]. Considering this unmet need and the barriers, this quality improvement (QI) initiative was conducted to increase the frequency of PPTL among multiparous women.

## Materials and methods

This QI was conducted at a tertiary care teaching hospital over a period of 18 months using a point of care QI methodology systematically. It was very disappointing to notice the high unmet needs of PPTL persisting among multiparous women. This QI aimed to combat the barriers to PPTL and increase its prevalence among multiparous women by 50% from baseline. The study consisted of three phases. Baseline observations during the pre-intervention phase over six months, intervention phase consisting of three Plan-Do-Study-Act (PDSA) cycles over 12 months, and post-intervention surveillance phase over a period of three months.

**Figure 1:**
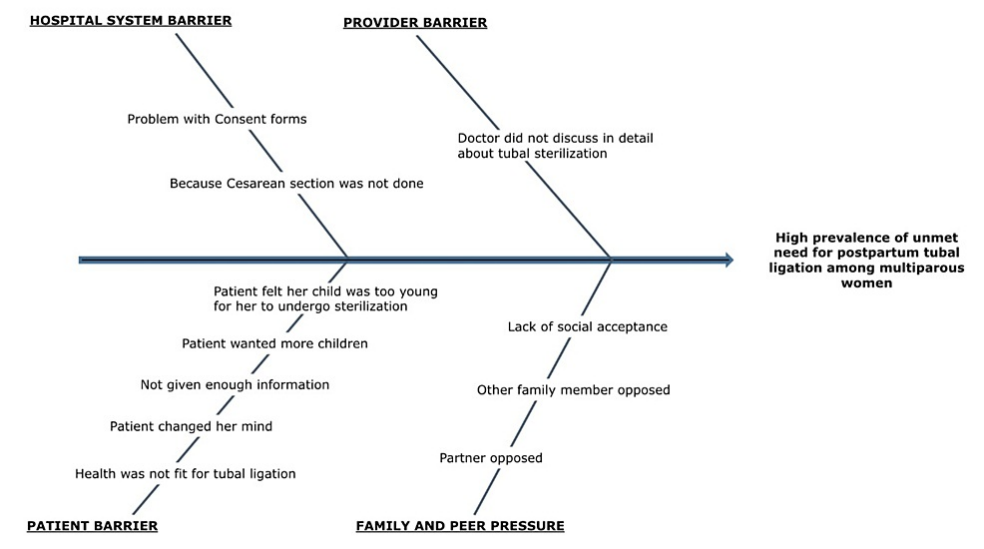
Fishbone analysis of the reasons for the increased prevalence of unmet needs of postpartum tubal ligation in the studied population

A baseline audit was performed to assess the prevalence of unmet needs of PPTL among multiparous women in the labor rooms and postpartum wards. A QI team was constituted and team members comprised faculty guides, resident doctors, and counselors working regularly in the antenatal clinics and labor wards. The dedicated QI team formulated the aim statement and designed the strategies for conducting the root cause analysis of the problem. Labor room and postpartum ward staff were inquired about the cause of such a high prevalence of unmet needs of PPTL. Designated team members from each duty shift were assigned the responsibility to carefully note the rate of PPTL requisition and its performance. Baseline data was observed over six months. A team meeting was conducted after the collection of the baseline observation in which the possible barriers to PPTL were discussed in detail. A conceptual framework of reasons for the increased prevalence of unmet needs of PPTL was devised in the form of a fishbone analysis (Figure 1). The Standard Operating Procedures of tubal ligation counseling for the antenatal mother, partner, and birth attendant were constituted after a detailed group discussion. For the husbands who did not accompany their wives to the antenatal clinics, counseling was done in labor wards after admission. Intervention plans were devised through proposed PDSA cycles to achieve a goal of 50% improvement.

The intervention phase continued for 12 months. It was driven by addressing the problems identified in the fishbone analysis. As a pilot intervention in the first PDSA cycle, hospital barriers and provider barriers were focused on. Consent forms were modified, and a separate column was added for PPTL. All recruited patients had a special red sticker on their antenatal card so that resident doctors do not forget to ask about PPTL after admission to labor wards. Treating doctors were asked to counsel and give the option for PPTL to all multiparous women qualifying the inclusion criteria. During the second PDSA cycle, patient barriers were addressed. In antenatal clinics, counselors and resident doctors did a group discussion in which the importance of contraception and the advantages of the small family were discussed with pregnant women. They also discussed the national criteria, advantages and disadvantages of tubal sterilization. Barriers at the level of partner and family members were addressed during the third PDSA cycle. In the antenatal clinics, partners were counseled and in the labor wards and triage areas, birth attendants were counseled routinely to study the effect of socio-cultural values on postpartum tubal sterilization.

Each PDSA cycle lasted for four months. The primary outcome was measured in the form of a rate of increase in PPTL after each successive intervention. Data collectors reported the outcome parameters like the total number of PPTL desired and the number of PPTL performed during their duty shifts respectively. A follow-up observation was conducted over a period of four months post-intervention to assess the rate of PPTL requisition and the rate of performance. During this period, only observation was done without any new intervention. Team members kept collecting the monthly observational data and plotting the line chart regularly. Data were entered in a Microsoft Excel matrix and analyzed using SPSS 20.0.0 (IBM Corp., Armonk, NY). All categorical variables were expressed in percentages and frequencies. The rate of PPTL requisition and PPTL performed was tabulated on a monthly basis. The rate of increase in PPTL requisition and PPTL performed was depicted in percentages and plotted in a line chart from the start to the end of the study.

## Results

**Figure 2:**
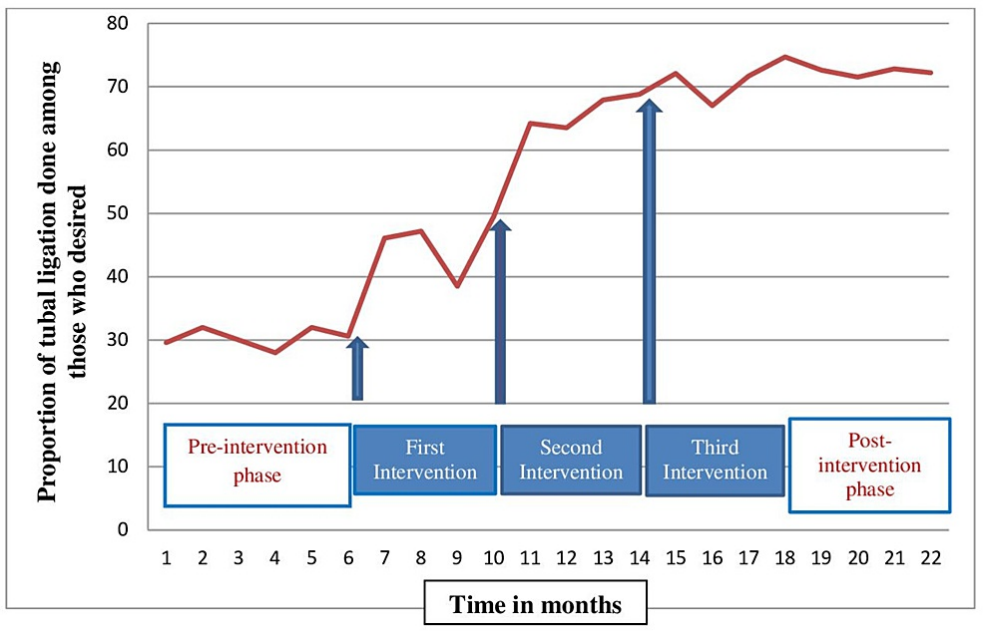
Run chart demonstrating the increase in postpartum tubal ligation after each intervention cycle in the studied population

A baseline audit demonstrated a low prevalence (30.2%) of PPTL in the studied population (Figure 2). Common reasons for this low prevalence were inadequate counseling and consent (due to heavy patient load), a procedure not being discussed by the doctors in detail, partner and family member opposition, and social nonacceptance [6]. The details of the data collection, recording, and analysis of the baseline audit were published previously [6]. So, the hurdles were at all levels like hospital barriers, service providers, patient and family barriers.

The prevalence of unmet needs for PPTL in the labor room and postpartum wards was tabulated on a monthly basis during each PDSA cycle. All observations were plotted in a line chart against time (Figure 2). During each intervention, there was an increase in the proportion of PPTL performed among the women who desired PPTL, as evident from Figure 2. In the first PDSA cycle, red stickers were pasted on the antenatal cards so that resident doctors and nursing staff did not forget to ask about PPTL after admission to labor wards. Doctors and nurses on duty were asked to counsel and give the option of PPTL to all multiparous women who were fulfilling the inclusion criteria. By the end of the first intervention cycle, the prevalence of PPTL performed increased from 30.2% to 49.5%. On further intervention during the second cycle in which counselors and resident doctors in the antenatal clinics did group discussion to explain about the importance of tubal sterilization, the advantage of small family norms and permanent methods of contraception, the prevalence increased to 68.8%. A further increase to 74.4% was observed after the third intervention which comprised partner counseling in the antenatal clinics or after admission to the labor room prior to delivery. During the follow-up observation period, an assessment was done to observe the consistency of the increase in PPTL performance. During this period, only observation was done with no new interventions. We observed that there was a satisfactory continuation rate of 72% at the end of the four-month follow-up. The multiparous patients whose desire for PPTL remained unfulfilled were advised and planned for interval tubal ligation. Among the patients who requested not to undergo PPTL after recruitment, all opted for postpartum intrauterine contraceptive devices. We propose to conduct six-monthly audits regularly to assess the impact of this QI initiative.

## Discussion

The permanent method of contraception is the most commonly used contraception method globally [7]. A qualitative study conducted on midwives stated that 50% of patients desired tubal ligation [3]. PPTL is considered safe, effective, and convenient for multiparous women [8]. Though it can be done at any time, the postpartum period would be better as the patient is in the hospital and postpartum is the period when she gets family support at the maximum. It is complex for women to decide regarding PPTL, particularly in developing countries like India because of the poor educational status of the women leading to a lack of knowledge, awareness and acceptance of tubal sterilization. Studies conducted to address the barriers to PPTL among multiparous women observed the hurdles at all levels including hospital, provider, patient, family and peer groups [6].

The barriers were problems with the process of counseling and obtaining consent, lack of operation theaters, doctors not discussing the procedure, change in the mind of the women, women desiring more children, social non-acceptance, and opposition from partners and family members [6]. To date, there were no QI initiatives conducted to improve the unmet need of PPTL. A QI initiative conducted to improve long-term contraception in postpartum women in Haiti proved successful by increasing the uptake from 5% to 32% [9]. Considering the effectiveness of this QI initiative in long-term contraception in postpartum and with the background of barriers to PPTL addressed in the earlier studies, we conducted the QI to improve the unmet need for PPTL. At the hospital level problems with the process of counseling and obtaining consent is one among the major barriers. Numerous analyses, strict administration, and regular audits can ensure a change in our attitude toward patient care and remove the barriers that can affect the unmet need for PPTL [10]. In the index study, few modifications were done in the process of counseling. Separate counseling sessions were organized in the antenatal clinics and beside the labor wards. About 50% of the women who were reluctant initially changed their minds after counseling [9,11]. A key rectifying measure would be to have a clinician inquest over the contraception desire during the course of pregnancy as 50% of desired women did not have a detailed discussion with the service provider [12]. We assumed that this hurdle can be overcome with the proper counseling of the antenatal women with a detailed discussion by the resident doctors starting from the antenatal clinics to the labor wards. The same was implemented in the first PDSA cycle. The World Health Organization recommends the improvement of knowledge, attitude and practice of antenatal care providers to ensure the provision of postpartum contraception information and services [13,14]. In order to improve the knowledge among the care providers, a group discussion about the importance of PPTL, government incentives and criteria of PPTL was implemented in the second PDSA cycle. Few previous studies have highlighted the religious and cultural barriers to postpartum sterilization [15,16]. We could not stress this aspect in the present QI initiative. Opposition by partners and family members was stated as an important barrier to PPTL [6,17,18]. To address and overcome this issue, counseling of the partner and birth attendant was implemented in the third PDSA cycle.

Postpartum contraception is of paramount importance in stabilizing the ever-increasing population, especially in LMIC [19,20]. The strength of this project is that it was conducted over a longer duration. The QI team comprised antenatal counselors, nurses and resident doctors who worked together to implement the QI strategies successfully. It is the first QI initiative conducted to address the barriers of PPTL in LMIC. The study had a few limitations as well. It was a single-center study. The post-intervention phase comprised only four months of observation. Religious and cultural barriers to PPTL remained unaddressed. Future initiatives with a large-scale population of different ethnicity can help to lay down recommendations in time to come.

## Conclusions

This QI initiative has provided promising results over time. We observed that there was continuous motivation among the service providers to educate and counsel the women for PPTL. It was also observed that the unmet need for PPTL improved and sustained over the follow-up phase. Ultimately, we were able to address the low prevalence of PPTL through modifications in our hospital strategies.
